# The Prognoses of Young Women With Breast Cancer (≤35 years) With Different Surgical Options: A Propensity Score Matching Retrospective Cohort Study

**DOI:** 10.3389/fonc.2022.795023

**Published:** 2022-02-28

**Authors:** Pei Li, Lun Li, Bingqiu Xiu, Liyi Zhang, Benlong Yang, Yayun Chi, Jingyan Xue, Jiong Wu

**Affiliations:** ^1^ Department of Breast Surgery, Fudan University Shanghai Cancer Center, Shanghai, China; ^2^ Department of Breast Surgery, Key Laboratory of Breast Cancer in Shanghai, Fudan University Shanghai Cancer Center, Shanghai, China; ^3^ Department of Breast Surgery, The Second Xiangya Hospital of Cancer South China, Changsha, China; ^4^ Collaborative Innovation Center for Cancer Medicine, Shanghai, China

**Keywords:** young breast cancer, survival, propensity score matching, surgical options, breast-conserving surgery

## Abstract

**Background:**

Compared with older patients, young women with breast cancer (YWBCs) have a poorer prognosis and a higher risk of recurrence. Ages ≤35 years are independent risk factors for local recurrence of breast cancer. Surgery is the most important local treatment for YWBC, and there is still a lack of prospective studies comparing surgical options for recurrence and survival. We retrospectively compared the effects of surgical options on disease-free survival (DFS) and overall survival (OS) of YWBC at Fudan University Shanghai Cancer Center (FUSCC).

**Methods:**

YWBCs (age ≤35 years) who underwent surgery at FUSCC between 2008 and 2016 were retrospectively analyzed and divided into three groups according to surgical options: 1) breast-conserving surgery (BCS), 2) mastectomy alone (M), and 3) mastectomy with reconstruction (RECON). The DFS and OS outcome rates from the three surgical options were compared using the Kaplan–Meier method and Cox regression model. Propensity score matching (PSM) was also used to balance the baseline characteristics to eliminate selection bias.

**Results:**

A total of 1,520 YWBCs were enrolled with a median follow-up of 5.1 years, including 524 patients (34.5%) who underwent BCS, 676 patients (44.5%) who underwent M, and 320 patients (21.1%) who underwent RECON. The 5-year DFS rates were 96%, 87%, and 93%, respectively (*P* < 0.001); the 5-year OS rates were 98%, 94%, and 97%, respectively (*P =* 0.002). Multivariate Cox analysis showed that DFS and OS were significantly improved in patients undergoing BCS compared with those undergoing M, with hazard ratios (HR) of 0.448 (95% CI 0.276–0.728; *P =* 0.001) and 0.405 (95% CI 0.206–0.797, *P =* 0.009), respectively. After PSM, DFS and OS rates were significantly improved in patients undergoing BCS compared to patients undergoing M (DFS, *P =* 0.001; OS, *P =* 0.009); RECON was also improved compared to patients undergoing M in terms of DFS and OS, but the difference was not statistically significant (DFS, *P =* 0.164; OS, *P =* 0.130).

**Conclusions:**

The surgical options were independent factors affecting DFS and OS in YWBC, and the DFS and OS rates were significantly improved in the BCS group compared to those in the M group. BCS is preferred for early YWBC, and RECON is the best option for remodeling the body images of YWBC who do not have breast-conserving conditions.

## 1 Introduction

Breast cancer is the most common malignancy among young women, accounting for 22% of cancer fatalities in 2017 (1). The controversiality of the cutoff age for defining young women with breast cancer (YWBCs) is different between China and Western countries. For instance, the European Society for Medical Oncology (ESMO) uses a cutoff of <40 years old, while the consensus and guidelines in China define the cutoff as age 35 or younger. There is a significant age difference in the worldwide incidence of breast cancer: the average age of breast cancer diagnosis is 45–55 years in China (2), which is 10 years younger than that in Western countries. Moreover, breast cancer patients under the age of 40 account for less than 7% of all breast cancer patients in developed countries. YWBCs account for more than 10% of all breast cancer patients in China (3). To certain the reasonable cutoff value for defining YWBC, The Korean Breast Cancer Society analyzed 9,885 breast cancer patients and found that the risk of death from breast cancer rises dramatically among women under the age of 35 (4). There is no consensus on a cutoff age value for defining YWBC by Eastern and Western scholars, although some researchers regard 35 years as a reasonable age value. However, the stratification of age has been widely accepted by doctors for decision-making regarding diagnosis and treatment.

There are three surgical options for breast cancer treatment: 1) breast-conserving surgery (BCS), 2) mastectomy alone (M), and 3) mastectomy with reconstruction (RECON). M is the most important local treatment for breast cancer; randomized controlled studies, such as the NSABP B-06 (5) and Milan (6) trials, demonstrated that survival outcomes after BCS combined with radiotherapy are equivalent to those after M for early breast cancer. Moreover, some studies have shown that BCS compared to M not only improved esthetic outcomes but also may be associated with survival benefits in recent years (7, 8). A large cohort study was published in *Lancet Oncology* in 2016, which found that BCS combined with radiation resulted in improved 10-year overall survival (OS) as compared to mastectomy (9). However, several retrospective studies have found that age is an independent risk factor for local recurrence in patients who underwent BCS (10–12). A Japanese study found that age was an independent factor for predicting ipsilateral breast tumor recurrence (IBTR) (*P =* 0.047); when patients were aged 40 years or younger, the 10-year IBTR rate was 15.7%; this was 3.8% in those aged 41–50 and 2% in those aged over 50 (10). Previous studies reported that age 35 years or younger was an independent risk factor for local recurrence in patients who underwent BCS (11, 13). A recent large cohort study demonstrated that the survival outcomes of BCS were better than those of M, and BCS should not be regarded as equal to M (14). However, the study did not focus on YWBC. There is still a lack of prospective studies to explore whether BCS could improve YWBC’s survival outcomes compared to other surgical options (15). Based on the demographic characteristics of Chinese patients with breast cancer, patients who were 35 years old or younger were included in our study. We retrospectively compared the effects of the three surgical options on the disease-free survival (DFS) and OS rates of YWBCs at Fudan University Shanghai Cancer Center (FUSCC). Therefore, our research may provide evidence-based data on surgical options for YWBC and explore the potential factors of these surgical options in terms of differences in cancer survival.

## 2 Methods

The FUSCC Ethics Committee approved this study (050432). Written informed consent for the study was waived due to the retrospective nature of our study.

### 2.1 Patient Screening

**Figure 1:**
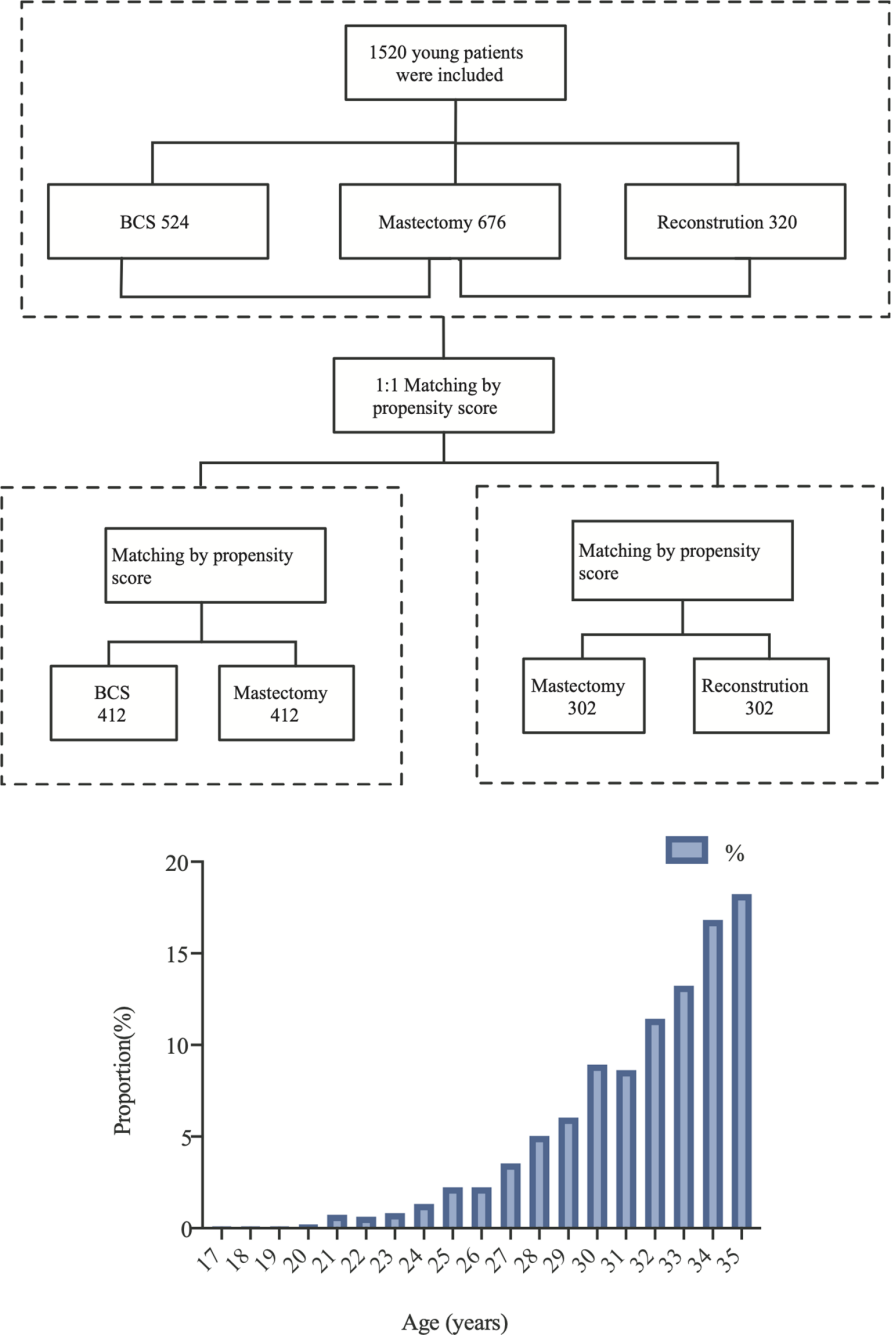
Flowchart and age composition ratio of included patients.

We retrospectively analyzed breast cancer patients who were inpatients at FUSCC for treatment between 2008 and 2016. The detailed inclusion criteria included: 1) primary and untreated breast cancer; 2) ages ≤35 years old; 3) patients who underwent surgery in our hospital and had no distant metastasis; 4) patients with Tis–T3 tumors according to the American Joint Committee on Cancer (AJCC) TNM stage system. The exclusion criteria included: 1) follow-up times shorter than 1 month; 2) patients with bilateral breast cancer or occult breast cancer; 3) patients who underwent neoadjuvant chemotherapy; and 4) lack of clinical data or follow-up data. A flowchart is shown in **Figure 1**.

### 2.2 Clinical Data Collection

YWBCs were identified from the FUSCC Breast Cancer Database. Two writers double-checked all of the information from the patients’ medical records (LP, LL). Prognostic data and follow-up information were provided by our breast cancer database.

**Figure 2:**
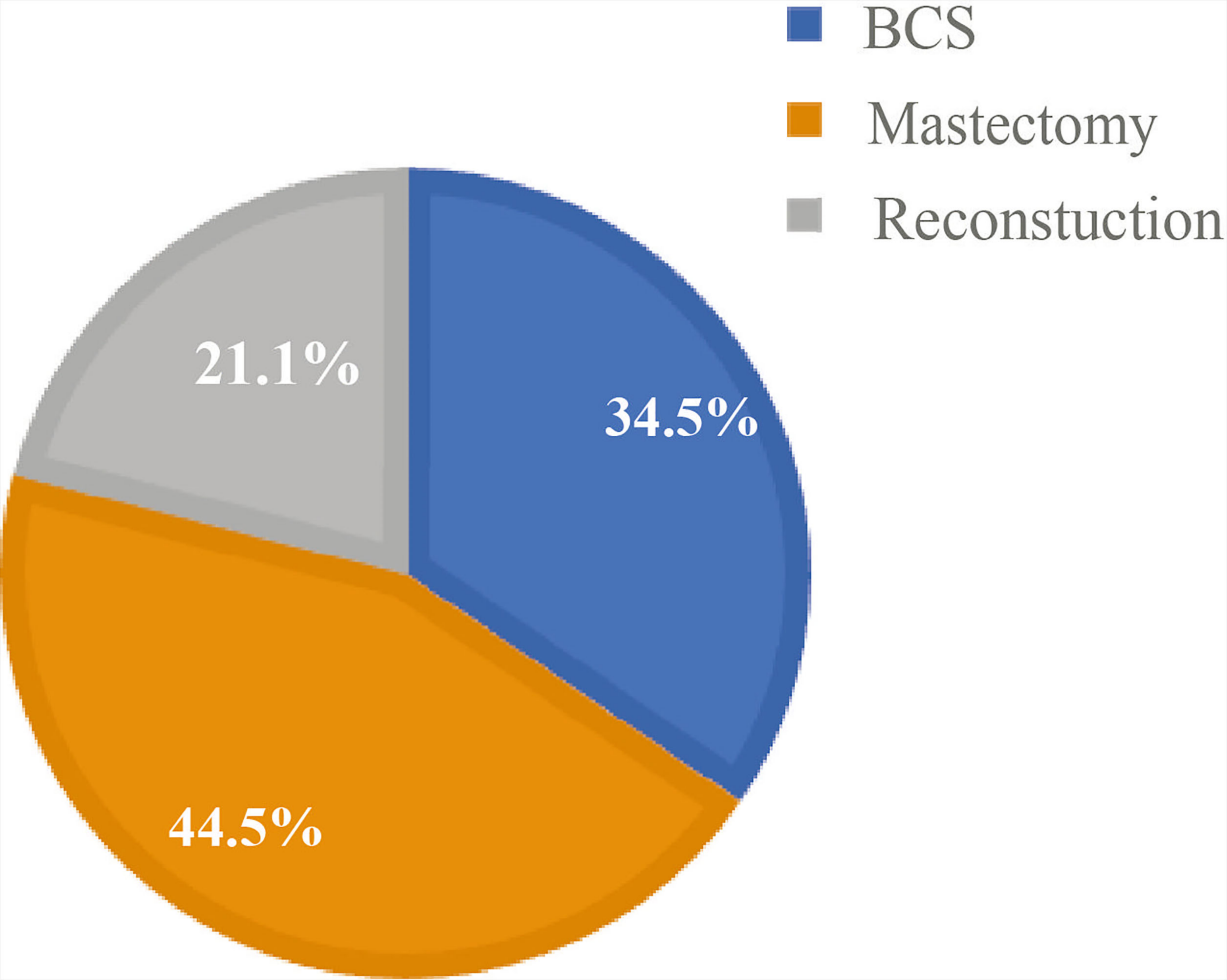
Percentage of surgical options in young patients at Fudan University Shanghai Cancer Center (FUSCC).

Study variables included patient age, body mass index (BMI), histological type, tumor grade, postoperative tumor size, lymph node metastasis status, estrogen receptor (ER) status, progesterone receptor (PR) status, and human epidermal growth factor receptor-2 (HER-2) status. The proportion of the patients included in this study is shown in **Figure 2**. BMI values were classified according to the criteria of the guidelines for the prevention and treatment of overweight and obesity in Chinese adults: normal, 18.5 ≤ BMI < 24; underweight, BMI < 18.5; and overweight, BMI ≥ 24. Oncological characteristics included histological type, such as ductal carcinom*a in situ* (DCIS), invasive ductal carcinoma (IDC), and others, as well as tumor grades classified as I, II, or III.

The clinical and pathological staging system of AJCC version 8 was used to evaluate patients’ T- and N-stage status (16). Hormone receptor-positive (HR, ER, or PR status) was defined as 1% expression by immunohistochemistry (IHC). HER2-positive breast cancer was defined as IHC staining 3+ or *ERBB2* gene amplification by fluorescence *in situ* hybridization (FISH). HER2-negative was defined as IHC staining 0 or 1+ or HER2 IHC staining 2+ and no gene amplification by FISH; triple-negative breast cancer (TNBC) was defined when ER status, PR status, and HER2 status were all negative. OS was calculated as the time from the initial pathological diagnosis to death from any cause as the clinical outcome assessment. DFS was defined as the time from the initial pathological diagnosis to the appearance of recurrence, metastasis, or breast cancer-related death. All patients were followed until the date of death or December 19, 2019. Patients lacking follow-up data were excluded from the study.

### 2.3 Propensity Score Matching

The R (version 4.0.4, https://www.r-project.org/) software was used for PSM using the “MatchIt” R packages. YWBCs who underwent surgery were divided into three groups according to surgical options: (1) BCS, (2) M, and (3) RECON. Survival outcomes were compared among the three surgical options using PSM to minimize the impact of selection bias and confounding variables. The variables included BMI, histological type, tumor grade, T stage, N stage, ER status, PR status, and HER2 status, as well as molecular subtypes. Patients were 1:1 matched using a caliper value of 0.5. The BCS vs. the M group had 412 patients after matching, and the M group had 302 patients (**Figure 1**).

### 2.4 Statistical Analysis

The baseline characteristics of the subgroup of surgical options were compared using Pearson’s chi-square test. DFS and OS were determined by Kaplan–Meier analysis and Cox regression model, and the survival outcomes of the three surgical options were compared using the log-rank test. A *P*-value <0.05 (95% confidence level) was considered statistically significant. All statistical analyses were conducted using SPSS (version 25.0; IBM Corporation, Armonk, NY, USA), and all survival curves were plotted using GraphPad Prism (Version 8.0; GraphPad Software, Inc., La Jolla, CA, USA).

## 3 Results

### 3.1 Characteristics of Patients

A total of 1,520 YWBCs were included in the study. The age composition is shown in **Figure 1**. The median follow-up duration was 5.1 years. A total of 524 patients (34.5%) underwent BCS, 676 patients (44.5%) underwent M, and 320 patients (21.1%) underwent RECON [**Figure 2**; ages, 31.02 (17–35), 32.23 (21–35), and 30.91 (19–35) years, respectively; **Table 1**].

**Table 1:** Baseline characteristics of young breast cancer patients with different surgical methods before propensity score matching.

Characteristic		Before PSM No. (%)			*P-value*
		BCS	Mastectomy	Reconstruction	
		N = 524	N = 676	N = 320	
Age	(average range)	31.02 (17~35)	32.23 (21~35)	30.91 (19~35)	
BMI					*P < 0.001*
	Normal (healthy weight)	382 (74.2)	460 (69.3)	221 (69.9)	
	Underweight	55 (10.7)	63 (9.5)	53 (16.8)	
	Overweight	78 (15.1)	141 (21.2)	42 (13.3)	
Histology type					*P < 0.001*
	DCIS	42 (8)	41 (6.1)	45 (14.1)	
	IDC	428 (81.7)	593 (87.7)	252 (78.8)	
	Other	54 (10.3)	42 (6.2)	23 (7.2)	
Grade					*P = 0.560*
	I, II	206 (51.4)	290 (54.2)	116 (55.5)	
	III	195 (48.6)	245 (45.8)	93 (44.5)	
pT					
	Tis	42 (8)	41 (6.1)	44 (13.8)	*P < 0.001*
	T1	239 (45.6)	260 (38.5)	145 (45.3)	
	T2	104 (19.8)	255 (37.7)	74 (23.1)	
	T3	0	22 (3.3)	10 (3.1)	
	NA	139 (26.5)	98 (14.5)	47 (14.7)	
pN					*P < 0.001*
	N0	366 (69.8)	346 (51.2)	227 (70.9)	
	N1	114 (21.8)	191 (28.3)	59 (18.4)	
	N2	20 (3.8)	88 (13)	20 (6.3)	
	N3	11 (2.1)	47 (7)	8 (2.5)	
	NA	13 (2.5)	4 (0.6)	6 (1.9)	
ER					*P = 0.660*
	Negative	145 (27.7)	185 (27.4)	80 (25)	
	Positive	378 (72.3)	490 (72.6)	240 (75)	
PR					*P = 0.660*
	Negative	162 (31)	224 (33.2)	107 (33.4)	
	Positive	361 (69)	451 (66.8)	213 (66.6)	
HER2					*P < 0.001*
	Negative	439 (83.8)	478 (70.7)	234 (73.1)	
	Positive	85 (16.2)	198 (29.3)	86 (26.8)	
Molecular subtypes					*P < 0.001*
	HR-/HER2+	20 (3.8)	82 (12.1)	33 (10.3)	
	HR+/HER2-	323 (61.6)	381 (56.4)	191 (59.7)	
	HR+/HER2+	65 (12.4)	116 (17.2)	53 (16.6)	
	TNBC	116 (22.1)	97 (14.3)	43 (13.4)	

BMI, body mass index; DCIS, ductal carcinoma in situ; IDC, invasive ductal carcinoma; NA, not available; ER, estrogen receptor; PR, progesterone receptor; HER2, human epidermal growth factor 2; TNBC, Triple-Negative Breast Cancer.

Before PSM, there were significant differences in BMI, histological subtype, T stage, N stage, and molecular subtypes among the three subgroups (**Table 1**). Analysis of the molecular subtypes showed that a larger proportion of TNBC patients underwent BCS as opposed to M and RECON (22% vs. 14% vs. 13%), while HER2-positive patients underwent BCS less frequently than M and RECON (16% vs. 29% vs. 27%) (**Table 1**). Compared with those who underwent BCS and RECON, a high proportion of patients who underwent M were overweight (21%), had T2 or T3 tumors (41%) and lymph node involvement (pN+, 48%), and were HER2-positive (29%). Compared with the other surgical options, patients who received RECON were mostly underweight (RECON vs. M vs. BCS, 17% vs. 10% vs. 11%, respectively), had ductal carcinoma *in situ* (RECON vs. M vs. BCS, 14% vs. 6% vs. 8%), and had negative lymph node involvement (RECON vs. M vs. BCS, 71% vs. 51% vs. 70%).

Our results demonstrated that the surgical options could be affected by the patients’ baseline characteristics. Therefore, patients were 1:1 matched to adjust for selective bias after PSM, with well-balanced BCS (n = 412) and M (n = 412) groups and with well-balanced RECON (n = 302) and M (n = 302) groups. After PSM, there were no differences between the matched groups in terms of their baseline matching variables (i.e., age, BMI, histology type and grade, T and N stages, ER status, PR status, and HER2 status) (**Tables 2**, **3**).

**Table 2:** After propensity score matching, the baseline characteristics of breast-conserving surgery vs. mastectomy alone.

Characteristic		Before PSM No. (%)		*P-value*	After PSM No. (%)		*P-value*
		BCS	Mastectomy		BCS	Mastectomy	
		N = 524	N = 676		N = 412	N = 412	
Age	(average range)	31.02 (17~35)	32.23 (21~35)		31.19 (18~35)	32.25 (21~35)	
BMI				*P < 0.001*			*P = 0.851*
	Normal (healthy weight)	382 (74.2)	460 (69.3)		291 (72.2)	299 (73.8)	
	Underweight	55 (10.7)	63 (9.5)		44 (10.9)	40 (9.9)	
	Overweight	78 (15.1)	141 (21.2)		68 (16.9)	66 (16.3)	
Histology type				*P < 0.001*			*P = 0.611*
	DCIS	42 (8)	41 (6.1)		41 (10)	33 (8)	
	IDC	428 (81.7)	593 (87.7)		342 (83)	348 (84.5)	
	Other	54 (10.3)	42 (6.2)		29 (7)	31 (7.5)	
Grade				*P = 0.560*			*P = 0.340*
	I, II	206 (51.4)	290 (54.2)		148 (35.9)	168 (40.8)	
	III	195 (48.6)	245 (45.8)		166 (40.3)	150 (36.4)	
pT				*P < 0.001*			*P = 0.706*
	Tis	42 (8)	41 (6.1)		41 (10)	34 (8.3)	
	T1	239 (45.6)	260 (38.5)		188 (45.6)	202 (49)	
	T2	104 (19.8)	255 (37.7)		104 (25.2)	97 (23.5)	
	T3	0	22 (3.3)		–	–	
	NA	139 (26.5)	98 (14.5)		79 (19.2)	79 (19.2)	
pN				*P < 0.001*			*P = 0.312*
	N0	366 (69.8)	346 (51.2)		267 (64.8)	278 (67.5)	
	N1	114 (21.8)	191 (28.3)		105 (25.5)	103 (25)	
	N2	20 (3.8)	88 (13)		20 (4.9)	19 (4.6)	
	N3	11 (2.1)	47 (7)		11 (2.7)	10 (2.4)	
	NA	13 (2.5)	4 (0.6)		9 (2.2)	2 (0.5)	
ER				*P = 0.660*			*P = 0.404*
	Negative	145 (27.7)	185 (27.4)		110 (26.7)	99 (24)	
	Positive	378 (72.3)	490 (72.6)		301 (73.1)	313 (76)	
PR				*P = 0.660*			*P = 0.398*
	Negative	162 (31)	224 (33.2)		125 (30.4)	114 (27.7)	
	Positive	361 (69)	451 (66.8)		286 (69.6)	297 (72.3)	
HER2				*P < 0.001*			*P = 1.000*
	Negative	439 (83.8)	478 (70.7)		333 (80.8)	333 (80.8)	
	Positive	85 (16.2)	198 (29.3)		79 (19.2)	79 (19.2)	
Molecular subtypes				*P < 0.001*			*P = 0.939*
	HR-/HER2+	20 (3.8)	82 (12.1)		20 (4.9)	20 (4.9)	
	HR+/HER2-	323 (61.6)	381 (56.4)		250 (60.7)	257 (62.4)	
	HR+/HER2+	65 (12.4)	116 (17.2)		59 (14.3)	59 (14.3)	
	TNBC	116 (22.1)	97 (14.3)		83 (20.1)	76 (18.4)	

PSM, propensity score matching; BCS, breast-conserving surgery; N, number; BMI, body mass index; DCIS, ductal carcinoma in situ; IDC, invasive ductal carcinoma; NA, not available; ER, estrogen receptor; PR, progesterone receptor; HER2, human epidermal growth factor 2; TNBC, Triple-Negative Breast Cancer.

**Table 3:** After propensity score matching, the baseline characteristics of reconstruction after total mastectomy vs. mastectomy alone.

Characteristic		Before PSM No. (%)		*P-value*	After PSM No. (%)		*P-value*
		Mastectomy	Reconstruction		Mastectomy	Reconstruction	
		N = 676	N = 320		N = 302	N = 302	
Age	(average range)	32.23 (21~35)	30.91 (19~35)		32.5 (21~35)	30.99 (19~35)	
BMI				*P < 0.001*			*P = 0.904*
	Normal (healthy weight)	460 (69.3)	221 (69.9)		216 (72.2)	218 (73.2)	
	Underweight	63 (9.5)	53 (16.8)		37 (12.4)	38 (12.8)	
	Overweight	141 (21.2)	42 (13.3)		46 (15.4)	42 (14.1)	
Histology type				*P < 0.001*			*P = 0.647*
	DCIS	41 (6.1)	45 (14.1)		35 (11.6)	42 (13.9)	
	IDC	593 (87.7)	252 (78.8)		243 (80.5)	239 (79.1)	
	Other	42 (6.2)	23 (7.2)		24 (7.9)	21 (7)	
Grade				*P = 0.560*			*P = 0.938*
	I, II	290 (54.2)	116 (55.5)		118 (39.1)	115 (38.1)	
	III	245 (45.8)	93 (44.5)		91 (30.1)	90 (29.8)	
pT				*P < 0.001*			*P = 0.510*
	Tis	41 (6.1)	44 (13.8)		34 (11.3)	41 (13.6)	
	T1	260 (38.5)	145 (45.3)		142 (47)	137 (45.4)	
	T2	255 (37.7)	74 (23.1)		69 (22.8)	71 (23.5)	
	T3	22 (3.3)	10 (3.1)		5 (1.7)	10 (3.3)	
	NA	98 (14.5)	47 (14.7)		52 (17.2)	43 (14.2)	
pN				*P < 0.001*			*P = 0.730*
	N0	346 (51.2)	227 (70.9)		222 (73.5)	210 (69.5)	
	N1	191 (28.3)	59 (18.4)		55 (18.2)	59 (19.5)	
	N2	88 (13)	20 (6.3)		14 (4.6)	20 (6.6)	
	N3	47 (7)	8 (2.5)		8 (2.6)	8 (2.6)	
	NA	4 (0.6)	6 (1.9)		3 (1)	5 (1.7)	
ER				*P = 0.660*			*P = 0.580*
	Negative	185 (27.4)	80 (25)		83 (27.5)	77 (25.5)	
	Positive	490 (72.6)	240 (75)		219 (72.5)	225 (74.5)	
PR				*P = 0.660*			*P = 1.000*
	Negative	224 (33.2)	107 (33.4)		102 (33.8)	102 (33.8)	
	Positive	451 (66.8)	213 (66.6)		200 (66.2)	200 (66.2)	
HER2				*P < 0.001*			*P = 0.645*
	Negative	478 (70.7)	234 (73.1)		224 (74.2)	219 (72.5)	
	Positive	198 (29.3)	86 (26.8)		78 (25.8)	83 (27.5)	
Molecular subtypes				*P < 0.001*			*P = 0.866*
	HR-/HER2+	82 (12.1)	33 (10.3)		31 (10.3)	32 (10.6)	
	HR+/HER2-	381 (56.4)	191 (59.7)		175 (57.9)	177 (58.6)	
	HR+/HER2+	116 (17.2)	53 (16.6)		47 (15.6)	51 (16.9)	
	TNBC	97 (14.3)	43 (13.4)		49 (16.2)	42 (13.9)	

PSM, propensity score matching; N, number; BMI, body mass index; DCIS, ductal carcinoma in situ; IDC, invasive ductal carcinoma; NA, not available; ER, estrogen receptor; PR, progesterone receptor; HER2, human epidermal growth factor 2; TNBC, Triple-Negative Breast Cancer.

### 3.2 Kaplan–Meier and Cox Analysis

#### 3.2.1 Disease-Free Survival

**Figure 3:**
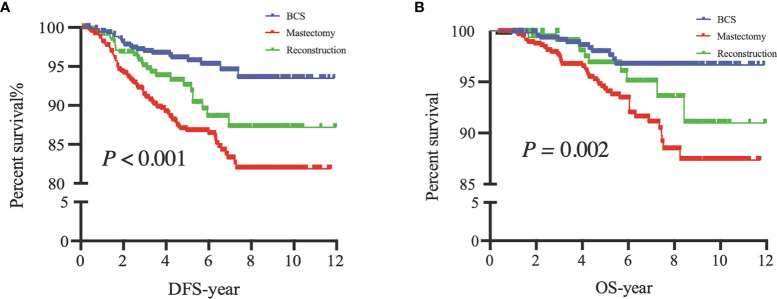
Disease-free survival (DFS) of patients among the three surgical options **(A)** and overall survival (OS) of patients among the three surgical options **(B)**. BCS, breast-conserving surgery; Mastectomy, mastectomy alone; Reconstruction, mastectomy with reconstruction.

The 5-year DFS rates were 96%, 87%, and 93% after BCS, M, and RECON, respectively; the 10-year DFS rates were 93%, 82%, and 87%, respectively, and the log-rank test showed a significant difference (*P* < 0.001) (**Figure 3**).

The multivariate Cox analysis showed that patients who underwent BCS had a significantly lower hazard of disease recurrences compared with those who underwent M [hazard ratio (HR) 0.441, 95% CI 0.274–0.709, *P =* 0.001], which could be an independent prognostic indicator for DFS. Compared to patients without lymph node metastasis, our results also showed that axillary lymph node involvement was an independent prognostic indicator of DFS (HR 1.661; 95% CI, 1.155–2.390; *P =* 0.006). BMI status, tumor size, histological type, grade, ER status, PR status, and HER2 status were not independent prognostic factors of DFS.

#### 3.2.2 Overall Survival

The 5-year OS rates after BCS, M, and RECON were 98%, 94%, and 97%, respectively; the 10-year OS rates were 97%, 87%, and 91%, respectively, and the log-rank test indicated a significant difference (*P =* 0.002) (**Figure 3**).

The multivariate Cox analysis showed that patients who underwent BCS had a significantly lower risk of death compared to those who underwent M (HR 0.461; 95% CI, 0.238–0.895; *P =* 0.022), which could be an independent prognostic indicator of OS. BMI status, tumor size, axillary lymph node status, histological type, grade, ER status, PR status, and HER2 status were not independent prognostic factors of OS.

#### 3.2.3 After Propensity Score Matching

**Figure 4:**
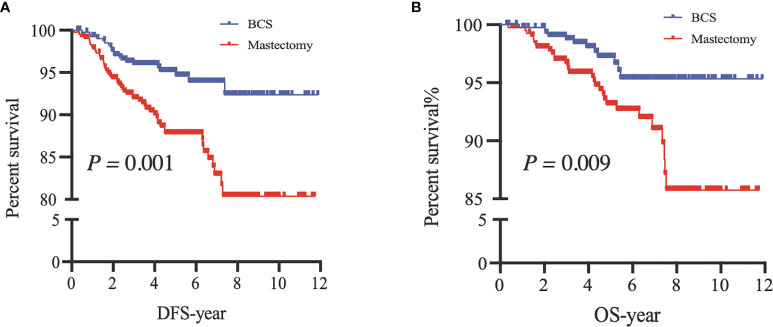
After propensity score matching, disease-free survival (DFS) **(A)** and overall survival (OS) **(B)** of patients between breast-conserving surgery (BCS) and mastectomy alone (Mastectomy).

**Figure 5:**
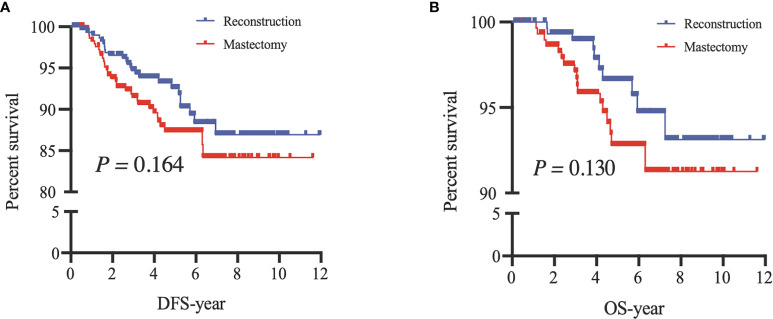
After propensity score matching, disease-free survival (DFS) **(A)** and overall survival (OS) **(B)** of patients between mastectomy with reconstruction (Reconstruction) and mastectomy alone (Mastectomy).

After PSM, our results based on the Kaplan–Meier and Cox analyses were consistent with those of the prematched results (**Figures 3**, **4**). The matching variables were BMI, histological type, tumor grade, postoperative pathological T stage, axillary N stage, ER status, PR status, HER2 receptor status, and molecular subtype. After PSM, DFS and OS rates were significantly improved in patients undergoing BCS compared with those undergoing M (DFS, *P =* 0.001; OS, *P =* 0.009; **Figure 4**), and the Cox analysis showed that BCS could improve DFS and OS [DFS: HR 0.378 (95% CI 0.227~0.630), *P* < 0.001; OS: HR 0.357 (95% CI 0.181~0.700), *P =* 0.003], which was consistent with the unmatched results. Patients who underwent RECON also showed improved DFS and OS rates compared with those who underwent M, but this difference was not statistically significant (DFS, *P =* 0.164; OS, *P =* 0.130; **Figure 5**).

## 4 Discussion

### 4.1 Study Findings

In our study, we compared the survival outcomes of different surgical options for YWBC; we found that the DFS and OS rates in the BCS group improved significantly in comparison to those in the M group, results that were similar to those seen in non-young patients. However, these results should be considered cautiously because the baseline characteristics and tumor burden of the patients between these two surgical options were significantly different. Some previous studies have shown that the selective bias of surgical options varies significantly depending on the institutions and surgeons (17, 18). In clinical settings, surgical decision-making for patients needs to incorporate age, family history, BMI, histological type and grade, TNM stage, molecular subtypes, and other special conditions. Thus, selective bias was unavoidable. Our results also demonstrated that the surgical options may be affected by the patients’ baseline characteristics. The patients who underwent M or RECON had a HER2 positive status, large tumor size (≥T2 stage), or more lymph node involvement (≥N1 stage) compared with those who underwent BCS (**Table 1**). Thus, PSM was used to adjust for confounding factors. After PSM, DFS and OS rates were significantly improved in the BCS group compared to those in the M group, and the RECON group also had improved rates compared to the M group; however, the improvements were not statistically significant (**Figures 4**, **5**).

### 4.2 Surgical Options and Systemic Therapy by Molecular Subtype

Several retrospective studies have demonstrated that age is an independent risk factor for tumor recurrence after BCS (10–12). The local recurrence of YWBC who underwent BCS could be reduced by systemic treatment in earlier studies, and the oncological outcomes of BCS combined with radiotherapy were regarded as being equal to M. With the advancement of systemic treatment, a recent large cohort suggested that BCS could improve survival outcomes compared to M, and these two surgical options should not be regarded as equal. A study in 2013 found that systemic therapy was associated with a nearly 60% lower incidence of local recurrence (HR 0.42; 95% CI 0.28–0.60; *P* < 0.0001) in YWBCs (aged ≤40 years) in the Netherlands, and distant relapse-free survival was not affected by late local recurrences (HR 1.24; 95% CI 0.74–2.08; *P* = 0.407) (19). A meta-analysis in 2015 summarized six studies that included 22,598 patients and showed that it appears unlikely that mastectomy provides a better OS than BCS in YWBC (≤40 years) (20). The rates of local and regional recurrence in YWBC (<35 years) were not affected by the surgical options. However, the recurrence varied by biomarker subtype, and when examined over the full study period (*P =* 0.056 and *P =* 0.014, respectively), these differences were borderline significant but leveled off after the introduction of trastuzumab after 2005 (*P =* 0.24 and *P =* 0.42, respectively) (21). However, it has been more than 10 years since these studies were conducted, and systemic therapy for breast cancer has developed rapidly in the past 10 years, especially in terms of precision treatment of molecular subtypes. Our results showed that the molecular subtypes were significantly different between the patients who underwent different surgical options (**Table 1**). Patients who underwent BCS were mainly the HR+/HER2- (62%) subtype that required adjuvant endocrine therapy (**Table 1**). The TEXT and SOFT trials found that ovarian function suppression plus tamoxifen or exemestane, instead of tamoxifen alone, significantly improved the 5-year breast cancer-free interval of YWBCs (<35 years) with HR-positive breast cancer (22). Our study included YWBCs (≤35 years old) between 2008 and 2016 and similarly found that patients who underwent BCS had improved DFS and OS outcomes compared to those who underwent BCS. This may be related to the advancement of precision treatment of molecular subtypes in recent years. Earlier studies on anti-HER2-targeted therapy have not been widely performed, and the times and intensities of endocrine therapy are different from those in the recent past. Molecular subtype markers have been transformed from prognostic markers to a therapeutic basis. Therefore, systemic therapy may play an essential role in reducing the recurrence and metastasis of BCS, thereby increasing the DFS and OS rates of YWBCs.

### 4.3 Surgical Options and Radiation Therapy as Well as Other Factors

The DFS and OS of YWBCs who underwent BCS were better than those who underwent M. All of these findings were based on adjuvant radiotherapy followed BCS. Moreover, the improved irradiation techniques for YWBCs play an important role in local recurrence. A randomized phase 3 trial (23) showed that the absolute probability of ipsilateral breast tumor recurrence was highly linked with the age of the patients. For individuals 35 years or younger, the 20-year cumulative incidence was 34.5%. However, a radiation boost followed by whole-breast irradiation (WBI) enhanced local control. The recurrences without or with boost irradiation were 13% and 9%, respectively, with the greatest absolute benefit in young patients. A review (24) summarized five randomized studies over a 10-year period to determine whether to receive a tumor bed boost or not after WBI and found that providing a boost resulted in a decrease in local recurrences while having no significant influence on other oncological outcomes. Therefore, tumor bed boost after WBI may be an effective factor for improvement of the DFS.

**Figure 6:**
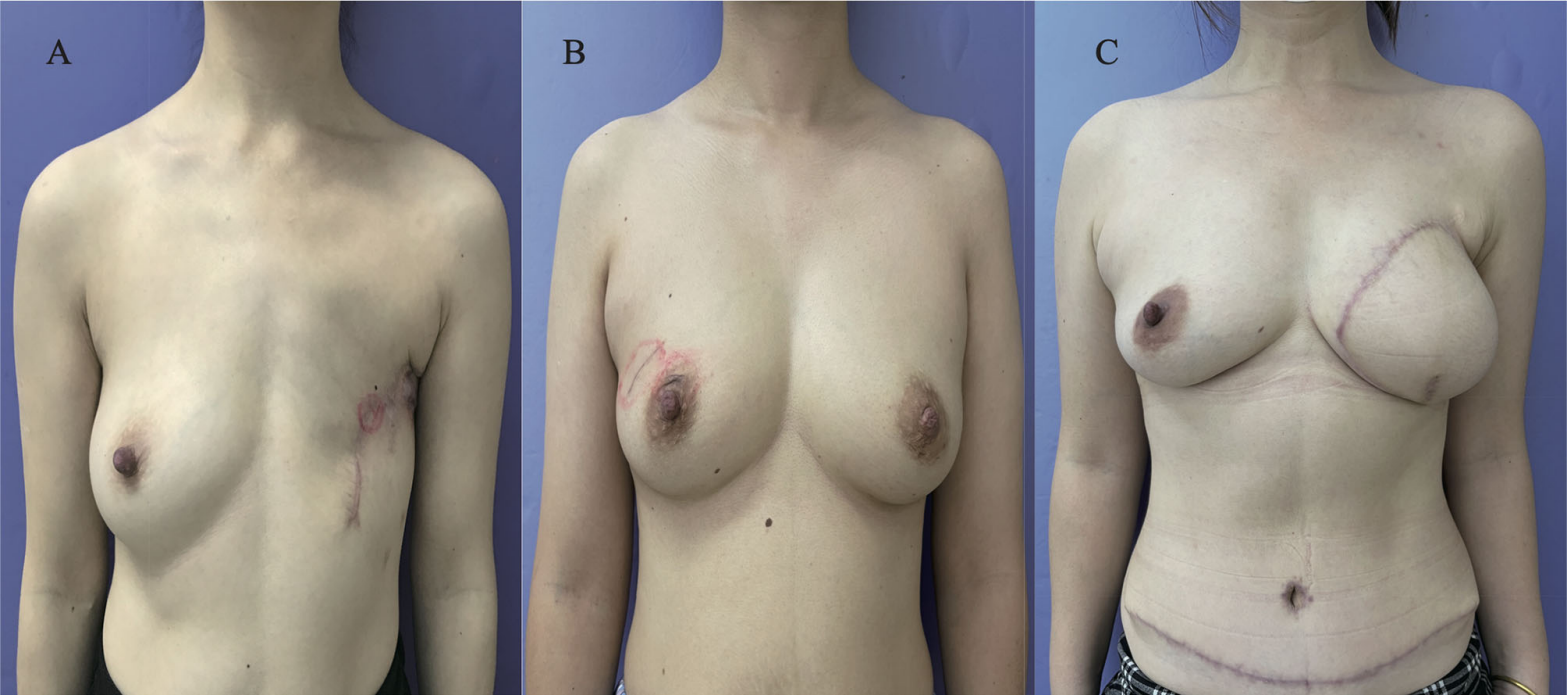
Postoperative esthetic results of patients from surgical options. **(A)** The esthetic images of patients who underwent mastectomy alone after 1 year; **(B)** the esthetic images of patients who had breast-conservation surgery after 6 months; **(C)** the esthetic images of patients who underwent mastectomy with reconstruction [deep inferior epigastric perforator flap (DIEP)] after 3 months.

The oncological safety of BCS is likely due to advances in systemic therapy, and optimal esthetics were achieved using BCS as opposed to M. RECON was the main method chosen to reshape the esthetics of the breasts in those who had contraindications to BCS. Local treatment of YWBCs, particularly those who underwent mastectomy, may have a long-term impact on breast satisfaction and psychosocial and sexual outcomes (25, 26). The DFS and OS rates of YWBCs who underwent BCS were better than those who underwent M, which may be due to improvements in systemic therapy and psychosocial factors; these findings warrant further investigation. Several studies found that the quality of life of the patients in the BCS and RECON groups was superior to that in the mastectomy group (27, 28). For instance, the YWBCs who underwent mastectomy had worse body images, sexual health, and anxiety than women who underwent less extensive surgery (24). Our patients’ esthetic results of three surgical options were consistent with those of other studies (**Figure 6**), BCS had greater breast satisfaction and quality-of-life ratings than RECON (29). However, there is no substantial evidence that BCS or RECON is superior. There were also other potential reasons for the improvements in DFS and OS results seen in patients who underwent BCS: the higher rates seen in BCS patients were linked to higher socioeconomic levels (14, 30), indicating that those patients were well educated and had higher incomes and health insurance. To summarize, the DFS and OS rates were significantly improved in patients who underwent BCS compared to those who underwent M, which may be a result of the patients’ quality of life or socioeconomic level.

## 5 Conclusion

The surgical options were independent factors that affected DFS and OS in YWBCs, and the DFS and OS rates were significantly improved in patients who underwent BCS compared to those who underwent M. This may be related to the development of systemic therapy and adjuvant radiotherapy to reduce the local recurrence of BCS. In addition, a complete body image could allow patients to return to their families and to society, as well as ensure a good quality of life. These findings warrant further investigation. Therefore, BCS is preferred for early YWBCs, and RECON is the best option for remodeling the body images of YWBCs who do not have breast-conserving conditions.

## Data Availability Statement

The raw data supporting the conclusions of this article will be made available by the authors, without undue reservation. Requests to access these datasets should be directed to 19111230031@fudan.edu.cn.

## Ethics Statement

The Fudan University Shanghai Cancer Center Ethics Committee approved this study (050432). Written informed consent from the participants’ legal guardian/next of kin was not required to participate in this study in accordance with the national legislation and institutional requirements.

## Author Contributions

PL, BY, and LZ collected the data. PL and LL planned and analyzed the study and wrote the paper. YC and BX assisted in the study. JX and JW revised the article. The final article was read and accepted by all contributors.

## Funding

Academic Leaders of Shanghai Science and Technology Commission funded this research (18XD1401300).

## Conflict of Interest

The authors declare that the research was conducted in the absence of any commercial or financial relationships that could be construed as a potential conflict of interest.

## Publisher’s Note

All claims expressed in this article are solely those of the authors and do not necessarily represent those of their affiliated organizations, or those of the publisher, the editors and the reviewers. Any product that may be evaluated in this article, or claim that may be made by its manufacturer, is not guaranteed or endorsed by the publisher.
